# A Combined Analysis of Gut and Skin Microbiota in Infants with Food Allergy and Atopic Dermatitis: A Pilot Study

**DOI:** 10.3390/nu13051682

**Published:** 2021-05-15

**Authors:** Ewa Łoś-Rycharska, Marcin Gołębiewski, Marcin Sikora, Tomasz Grzybowski, Marta Gorzkiewicz, Maria Popielarz, Julia Gawryjołek, Aneta Krogulska

**Affiliations:** 1 Department of Pediatrics, Allergology and Gastroenterology, Collegium Medicum in Bydgoszcz, Nicolaus Copernicus University in Torun, 87-100 Toruń, Poland; maria.popielarz@cm.umk.pl (M.P.); julia.gawryjolek@cm.umk.pl (J.G.); aneta.krogulska@cm.umk.pl (A.K.); 2 Department of Plant Physiology and Biotechnology, Nicolaus Copernicus University in Torun, 87-100 Toruń, Poland; 3 Interdisciplinary Centre of Modern Technologies, Nicolaus Copernicus University in Torun, 87-100 Toruń, Poland; sikoramar@op.pl; 4 Department of Forensic Medicine, Collegium Medicum in Bydgoszcz, Nicolaus Copernicus University in Torun, 87-100 Toruń, Poland; tgrzyb@cm.umk.pl (T.G.); gorzkiewiczmarta@cm.umk.pl (M.G.)

**Keywords:** atopic dermatitis, dysbiosis, food allergy, gut, infants, microbiota, skin, 16S rRNA sequencing

## Abstract

The gut microbiota in patients with food allergy, and the skin microbiota in atopic dermatitis patients differ from those of healthy people. We hypothesize that relationships may exist between gut and skin microbiota in patients with allergies. The aim of this study was to determine the possible relationship between gut and skin microbiota in patients with allergies, hence simultaneous analysis of the two compartments of microbiota was performed in infants with and without allergic symptoms. Fifty-nine infants with food allergy and/or atopic dermatitis and 28 healthy children were enrolled in the study. The skin and gut microbiota were evaluated using 16S rRNA gene amplicon sequencing. No significant differences in the α-diversity of dermal or fecal microbiota were observed between allergic and non-allergic infants; however, a significant relationship was found between bacterial community structure and allergy phenotypes, especially in the fecal samples. Certain clinical conditions were associated with characteristic bacterial taxa in the skin and gut microbiota. Positive correlations were found between skin and fecal samples in the abundance of *Gemella* among allergic infants, and *Lactobacillus* and *Bacteroides* among healthy infants. Although infants with allergies and healthy infants demonstrate microbiota with similar α-diversity, some differences in β-diversity and bacterial species abundance can be seen, which may depend on the phenotype of the allergy. For some organisms, their abundance in skin and feces samples may be correlated, and these correlations might serve as indicators of the host’s allergic state.

## 1. Introduction

Although the cause of the growing prevalence of allergic diseases remains unclear, the Old Friends Hypothesis (also called the Biodiversity Hypothesis) has recently been proposed [1]. The hypothesis states that the development of many diseases, including allergy, might be attributed to a lack of exposure to the “right” bacterial strains. Assuming this to be the case, it may be possible that identifying such bacteria could halt the allergic march and foster the development of novel prevention and treatment methods for allergic diseases. 

The composition of the human microbiome is not only characteristic of each individual, but also evolves over time [2,3,4,5,6,7]. In various parts of the child’s body, the microbiome begins to form and differentiate within the first six weeks post natum [2,3]. The most pronounced changes in the gut microbiome are usually observed during first two or three years of human life [4,5,8]. Following this, the composition of the gut microbiome remains relatively stable, unless significant environmental changes occur [5]. The early gut microbiome is believed to be shaped by a range of factors, including maternal race-ethnicity, age, diet, antibiotic treatment, marital status, the mode of delivery, health complications after birth, infant feeding method, solid introduction, probiotics intake, exposure to tobacco smoke and pets [2,4,6,7].

Similarly, the skin microbiota evolves over the years according to changes in skin structure and function [9], becoming similar to that of adults by the age of 12–18 months [10]. A possible critical window during which microbial-based intervention would be possible is early infancy (i.e., the first six months of life) [10,11,12]. The composition of skin microbiota is site-specific and, again, depends on a range of factors, including local conditions such as invaginations, pockets, niches and surfaces, as well as moisture, sebum secretion, temperature and exposure to external factors [9,10]. In the neonatal period, the mode of delivery appears to play an important role in shaping the skin microbiome [2]. The microbiome, considered a complex biocenosis where various organisms interact, influences the immune system in a multifaceted way, modulating both innate and adaptive immunity, and shaping allergy development through interactions with the host genome [13,14,15]. 

The gut and the skin are crucial barriers through which environmental factors interact with the human body; the two compartments are also massively colonized with distinct microbial communities. Furthermore, they constitute complex immune organs that are fully integrated into the overall immune system. Proper skin and gut functioning is essential for homeostasis, and decreases the risk of allergy. Hence, exposure to the many environmental factors that shape the microbiome of the gut and skin epithelium can alter the epithelial surfaces and thus drive the type 2 immune response, which is known to underlay most, if not all, atopic diseases. Many studies indicate the existence of a gut–skin axis, which is modulated by microbiota and its metabolites [16,17,18]. 

Generally, the first symptoms of allergy involve the skin and/or the gastrointestinal tract [19], manifesting as atopic dermatitis (AD) and/or food allergy (FA). These disorders are difficult to diagnose in early infancy, as there are no specific markers, and diagnosis is based mainly on their symptoms. Although previous studies have yielded varying results, it has been found that gut microbiota differed between otherwise healthy subjects and those who were food sensitized (FS) or food allergic (FA) [20,21]. It has been shown that the composition of the microbiota in the neonatal period may be associated with the development of allergies up to the age of one year. In later life, alpha-diversity, beta-diversity, and the richness of individual bacteria are shaped differently in non-allergic and allergic children [3]. It has also been shown that the microbiome structure at three months is associated with the development of sensitization up to one year of age [11]. It also appears that the microbiome of the gut in infancy is a prognostic factor of allergy resolution by eight years of age [12]. 

The bacterial community structure has also been found to differ between IgE-mediated and non-IgE-mediated FA children [22], and differences can be seen between the microbiota associated with specific food allergies [23,24,25]. Fieten et al. report no differences in microbial diversity between FA and non-FA children; however, a fecal microbial signature that discriminated between the presence and absence of FA was found in children with AD [26]. 

Similarly to the gut microbiome, the skin microbiota has also been found to differ between patients with AD and those without [9], and other studies have reported decreased skin microbiome diversity in AD patients [16,27]. The significance of the skin microbiome in AD pathogenesis was confirmed by Laborel-Preneron et al., who showed that *Staphylococcus aureus* colonizing atopic skin promoted inflammation in children aged one to three years, while commensal *S. epidermidis* strains might mitigate this effect [28]. Similarly, Kong et al. found that the abundance of *Staphylococcus* (particularly *S. aureus*) was greater in children with AD, and that microbial diversity increased over the course of treatment [29]. Interestingly, while the development of AD appears to be related to colonization by *S. aureus*, it has also been proven that exacerbations of skin lesions are connected to a reduction in skin microbiome diversity, while improvement is not directly related to a reduction in *S. aureus*, but rather to an increase in bacterial diversity [16]. In addition, prospective studies have identified changes in gut and/or skin microbiota in children, even prior to the development of food sensitivity (FS), FA or AD, with changes being apparent as early as in the third month of life [11,16,30,31,32,33]. It has also been proposed that decreased *Staphylococcus* richness in the skin microbiome during early infancy might be a prognostic factor of AD development before 12 months of age, and that colonization by commensal *Staphylococci* might protect against eczema [32]. 

Current evidence suggests that while the gut microbiota may demonstrate a reduction in microbial diversity before AD onset, no such relationship has been noted for the skin microbiota [16,23]. Furthermore, although gut dysbiosis has been described in AD [23,27,34], it is not clear whether the modulation of gut microbiota can impact skin microbiota and vice versa. The role of the skin microbiome in FA development is also unknown. 

To gain a more holistic understanding of how the microbiome of the human body interacts with the immune system, and how it can influence the development of FA and/or AD, it is necessary to study the skin and gut together as part of one combined study. The present study is the first such study to compare the microbiota of two compartments, i.e., gut and skin, in young infants with early onset of FA and/or AD. Indeed, of all the studies regarding the relationship between microbiota and allergy published to date, only one [35] has analyzed multiple compartments.

The present study characterizes the alpha and beta diversity of the fecal, i.e., gut, and skin bacterial communities from infants with FA and/or AD and compares them with those of healthy control subjects. 

We hypothesize that:

**Hypothesis** **1.** *Gut and skin alpha diversity differ between children with an allergy (FA and/or AD) and those without;*

**Hypothesis** **2.** *The communities within a single compartment differ according to the clinical status of the child;*

**Hypothesis** **3.** *OTUs characteristic for each compartment-clinical status communities exist;*

**Hypothesis** **4.** *Relationships exist between the gut and skin microbiota, and these differ with regard to clinical status.*

To confirm these hypotheses, V3-V4 16S rRNA gene libraries were generated and sequenced, and the resulting sequences were analyzed bioinformatically. 

## 2. Methods

This is an observational pilot study, constituting part of Stage I of a prospective study.

### 2.1. Study Group

Participants were recruited from 1 February 2018 until 30 January 2019 among infants hospitalized in the Department of Pediatrics, Allergology and Gastroenterology, Collegium Medicum, Nicolaus Copernicus University, Poland and from Gastrological, Allergological and Nephrological Outpatient Clinics in Bydgoszcz, Poland. 

**Figure 1:**
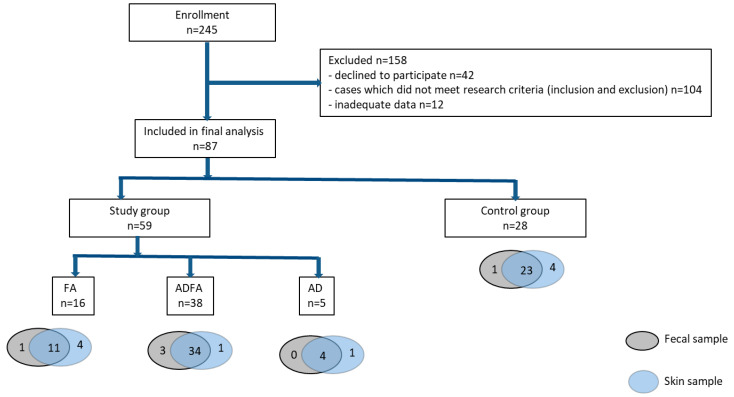
Study design. Flow chart depicting steps involved in patient selection and tests in this study.

Enrollment was proposed to parents of 245 infants under six months of age. Informed consent was obtained from 203 of them. Out of these patients, 104 did not fulfill the inclusion criteria, or were excluded (Table 1), and the parents of 12 failed to provide the full data needed for the study. The primary cause of disqualification from the study was receipt of known bacterial, viral or fungal infection or antibiotic treatment, either at the present time or in the previous month, due to their impact on the microbiome. The recruitment procedure is summarized in Figure 1. 

Finally, 87 infants were enrolled, 59 of them with suspicion of allergies (henceforth called the “allergic group” or “study group”) and 28 healthy infants, comprising the control group. The allergic group comprised 38 patients with symptoms of FA and AD (ADFA group), 16 with symptoms of FA (FA group) and 5 with AD (AD group). 

The characteristics of the study and control group are given in Table 2. No significant differences were observed between the FA/AD, FA, AD and control groups in terms of cesarean section, diet (breastfeeding or formula-feeding and solids intake) or family history of allergic diseases. The clinical characteristics of the studied infants are given in Table A1. In the studied group, FA was equivalent to cow’s milk allergy (CMA). IgE-sensitization was found in eight (14.8%) infants.

### 2.2. Preliminary Questionnaire

The participants completed the modified ISAAC questionnaire [36]. It comprised questions on the family history of atopic disease, as well as the presence of symptoms in the child and their relationship with diet, perinatal conditions, social background and demographic data. 

### 2.3. FA Diagnosis

The diagnosis of FA was based on history, physical examination, COMISS evaluation, specific IgE in the blood, elimination and oral provocation tests.

COMISS (Cow’s milk-related symptoms score) was assessed based on the standard questionnaire [37]. Scores ≥ 12 were regarded as indicating a high probability of FA. Other factors taken into consideration were: (i) in breastfed children: remission on elimination diet and relapse on reintroduction of milk, according to EAACI [38]; in children fed with milk formula: remission on elimination diet and positive outcome of open oral food provocation test according to ESPGHAN and EAACI [19,39], (ii) typical symptoms related to cow’s milk protein intake (frequent regurgitation or vomiting; extended periods of diarrhea with negative microbiological tests; blood in stools; chronic poor weight gain on milk inclusion; iron deficiency anemia due to cryptic or macroscopic blood loss with stools and not due to infection or insufficient iron intake); eosinophilic enteropathy confirmed with endoscopy; running nose, wheezing and chronic coughing unrelated to infection; atopic eczema (moderate or severe, defined as persistent or frequently recurring eczema with typical morphology and distribution, requiring frequent need for prescription topical corticosteroids, calcineurin inhibitors, despite appropriate use of emollients.); urticarial (i.e., unrelated to infections, drug intake and other causes) swelling of skin or hives; recurrent itchy or flushed skin; anaphylaxis [19].

### 2.4. Atopic Dermatitis (AD) Diagnosis

Atopic dermatitis (AD) was diagnosed based on the UK Working Party’s criteria with modifications necessary for infants [40,41], i.e., itchy skin (or parental report of scratching or rubbing) and at least three of the following symptoms:

- history of skin rash affecting the flexures (folds of elbows, behind knees, fronts of ankles), cheeks, neck, around eyes or outer surfaces of limbs;
- history of atopic diseases in a first-degree relative;
- history of generally dry skin;
- visible eczema involving cheeks, forehead, flexures and extensor/outer surfaces of limbs.

AD severity was assessed based on SCORing Atopic Dermatitis (SCORAD) scale at study entry [42]. AD patients were further stratified into two groups based on the presence of accompanying FA symptoms:

- AD without FA;
- AD with FA, i.e., eczema despite emollients; eczema and other typical symptoms of FA; significant improvement after elimination diet and deterioration after reintroduction of presumed allergen into the diet.

### 2.5. Sample Collection

Fecal and skin samples were collected once, during the visit at the time of study enrollment. Sterile, disposable equipment was used throughout the sampling procedure. Freshly evacuated feces in diapers were gathered into collection tubes and frozen for transport to the laboratory. Skin swabs were collected from non-lesional, healthy looking skin on the cheeks; in cases where the cheeks were atopic, sampling was performed from any other healthy skin region excluding the diaper area, usually from the dorsal surface of the forearm. All of the samples were stored at −80 °C until further processing.

### 2.6. Determination of sIgE

Serum IgE was quantified with Polycheck test (BioCheck GmbH, Leipzig, Germany) according to the manufacturer’s protocol. The detection limit was assessed as 0.35 kU/l. The patient was regarded as sensitized if specific IgE (sIgE) concentration exceeded the detection limit. Main food allergens (cow’s milk, hen’s egg, peanuts, soybean, wheat, hazelnut, codfish) and inhalant ones (house dust mites *Dermatophagoides pteronyssinus* and *D. farinae*, cat dander, dog dander, birch, hazel, and grass pollen, olive and Alternaria spores) were tested.

### 2.7. Metagenomic Analysis 

DNA was isolated from clinical samples using bead-beating (on PowerLyzer, MoBio) combined with flocculation and silica columns. V3-V4 bacterial 16S rRNA gene fragments were amplified and converted into Illumina sequencing libraries as previously described [43]. The libraries were sequenced on MiSeq using 600 cycles v.3 kit (Illumina) at CMIT NCU.

### 2.8. Bioinformatics and Statistical Analyses 

The sequencing reads were processed as described earlier [43]. Briefly, the reads were denoised, merged and checked for the presence of chimaeras in dada2 [44]; they were then classified using SILVA v.132 reference [45]. Following this, OTUs were constructed, and a shared OTU table was calculated with Mothur [46]. Ecological analyses were performed in R using functions implemented in the vegan [47] and GuniFrac packages [48,49]. An unweighted UniFrac distance matrix was generated using the GuniFrac function from the rarefied OTU table and tree using the Relaxed Neighbor Joining algorithm implemented in clearcut [50]. Unconstrained ordination was performed using non-metric multidimentional scaling (NMDS), implemented in the metaMDS function of vegan. The grouping significance was tested with PERMANOVA (adonis function) using 999 permutations; dbRDA was performed on the unweighted UniFrac distance matrix using dbrda. The significance of the dbRDA model was tested with anova.rda using 999 permutations. *p*-value < 0.05 was considered significant. 

Differences in taxa abundance were assessed with ANOVA (aov function in R); the normality of data was checked with the Shapiro–Wilk test (shapiro.test) and homogeneity of variance by Levene’s test (levene.test of the lawstat packege). Where the assumptions were not fulfilled, the Kruskal–Wallis test was used (kruskal.test). *p*-values were corrected for multiple comparisons using Benjamini-Hochberg FDR (*p*.adjust); a significance level of 0.05 was used. Clinical categorical data were analyzed using two-tailed Fisher’s exact test in R (fisher.test); again, a significance level of 0.05 was used.

### 2.9. Additional Information

All methods were carried out in accordance with relevant guidelines and regulations. The study protocol was approved by the local Ethics Committees of the Institutional Review Board of CM Bydgoszcz NCU Torun, Poland (765/2017). Informed written consent was obtained from the parents of all participants prior to enrollment.

### 2.10. Data Availability

Sequence data generated during this project are available in the SRA database under BioProject no. PRJNA657878.

## 3. Results

### 3.1. No Significant Differences in Alpha Diversity Indices 

**Figure 2:**
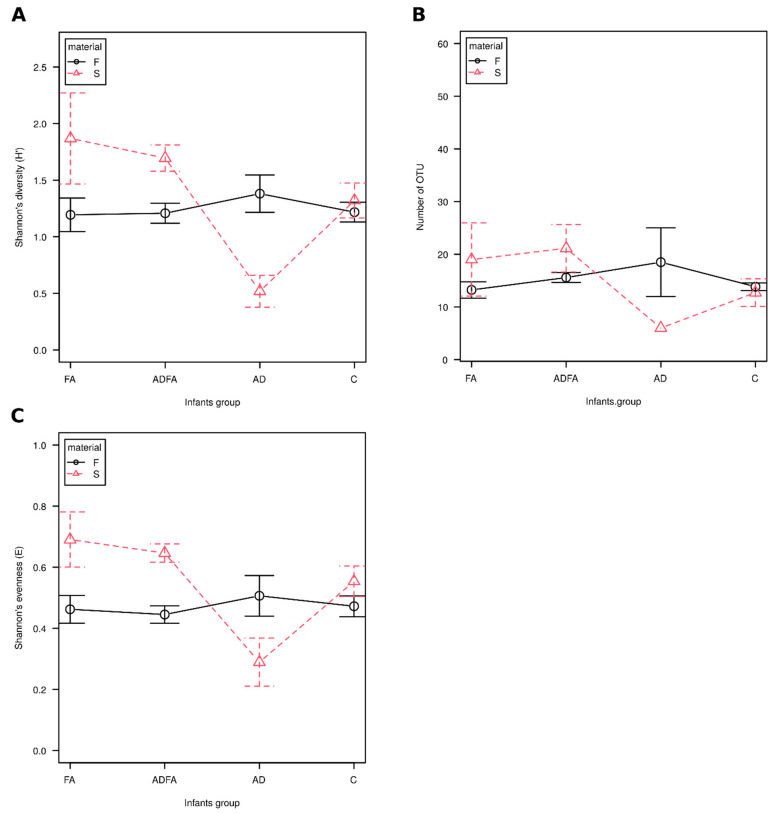
α-diversity of feces and skin microbiota in infants with FA, AD and ADFA. F—feces, S—skin, FA—food allergy, AD—atopic dermatitis, ADFA—atopis dermatitis and food allergy, C—control group. (**A**) Shannon’s diversity index (H’), (**B**) Observed number of OTUs, (**C**) Shannon’s evenness (E).

No significant differences in diversity (Shannon’s H’), species richness (observed number of OTUs) and evenness (Shannon’s E) were found between controls and the participants with either AD, ADFA or FA, regardless of the compartment (skin and gut). Similarly, no differences were found between the group of allergic patients and those without an allergy. However, the diversity of skin microbiota tended to be lower in AD patients (Figure 2). No correlations of any alpha-diversity indices were observed between the skin and feces, regardless of the analyzed group.

### 3.2. Gut and Skin Microbiota Differ According to Clinical Status

**Figure 3:**
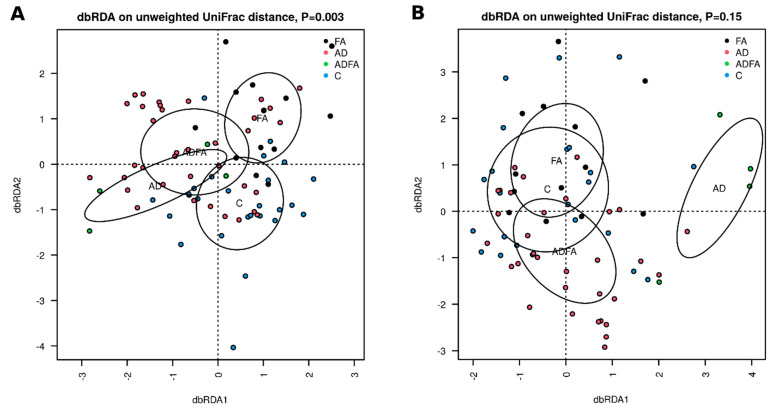
(**A**,**B**). β-diversity of fecal and skin microbiota in the studied groups. β-diversity: distance based redundancy analysis (db RDA) on unweighted distance metrices calculated on rarified community data for: A—feces, B—skin, 95% confidence elapses for show significance of grouping according to clinical status was tested FA—food allergy group, AD—atopic dermatitis group, ADFA—atopic dermatitis and food allergy group, C—control group.

Significant differences were observed between the control, AD, ADFA and FA groups regarding fecal sample community structure (unweighted UniFrac distance, dbRDA, *p* = 0.003; Figure 3A). In addition, differences were found between ADFA and FA (*p* = 0.04) and between ADFA and control (*p* = 0.05). 

Although no significant overall differences in skin communities were observed between groups (*p* = 0.15), significant differences became apparent when age (treated as continuous variable) was added to the overall model (*p* = 0.013). Regarding individual group comparisons, significant differences in the skin microbiome were found between AD and FA (*p* = 0.014), AD and control (*p* = 0.01), as well as AD and ADFA (*p* = 0.02) (Figure 3B). 

### 3.3. There Are Taxa Characteristic for Compartments and Clinical Status

**Figure 4:**
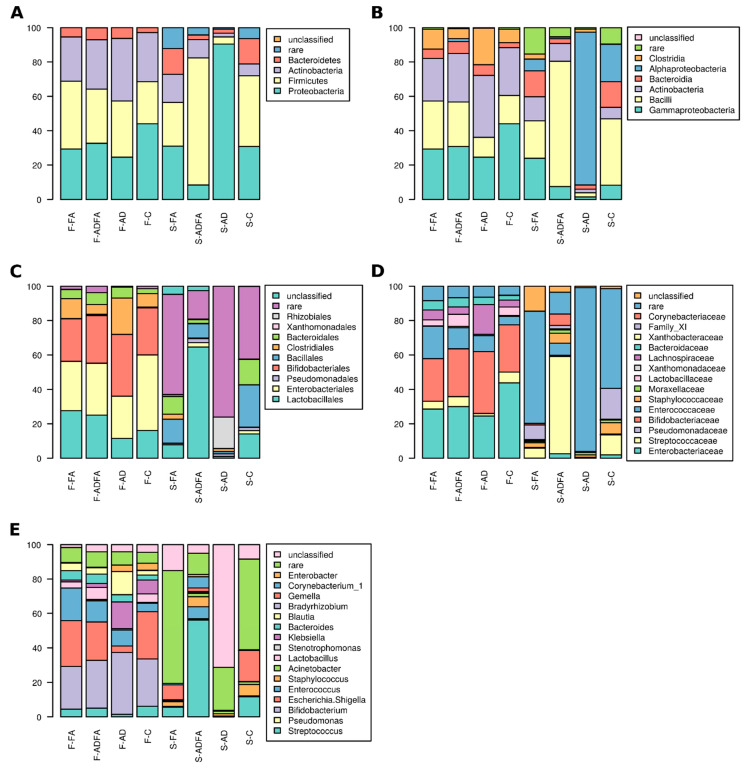
Taxonomic composition: (**A**). phyla level, (**B**). class level, (**C**) order level, (**D**). family level, (**E**). genus level in feces and skin microbiota of the studied children. F-FA—feces in food allergy group, F-AD—feces in atopic dermatitis group, F-ADFA—feces in atopic dermatitis and food allergy group, F-C—feces in control group, S-FA–skin in food allergy group, S-AD—skin in atopic dermatitis group, S-ADFA—skin in atopic dermatitis and food allergy group, S-C—skin in control group.

Any taxa whose abundance differed significantly between groups were regarded as characteristic for the group where the given taxon was more highly represented (Figure 4).

The members of the Xanthomonadales and Xanthomonadaceae were more abundant in the skin communities of all allergic (AD + ADFA + FA) patients, than in healthy children (*p* = 0.04 and *p* = 0.004, respectively); however, the reverse was true for the order Lactobacillales (*p* = 0.045), family Streptococcaceae (*p* = 0.045), genus *Streptococcus* (*p* = 0.044), and OTU13 belonging to genus *Bradyrhizobium* (*p* = 0.019).

In the gut of allergic children, the following taxa were more abundant: phylum Bacteroidetes (*p* = 0.03), class Bacteroidia (*p* = 0.03), order Bacteroidales (*p* = 0.046), family Bacteroidaceae (*p* = 0.043) and genus *Bacteroides* (*p* = 0.043). At the OTU level, OTU35, affiliated with *Parabacteroides*, and OTU21, belonging to *Bacteroides,* were more abundant in allergic children (*p* = 0.039 and *p* = 0.008, respectively), while OTU94 (*Fusicatenibacter saccharivorans*; *p* = 0.049), OTU68 (*Lactococcus lactis*; *p* = 0.035) and OTU30 (*Serratia marcescens*; *p* = 0.035) were more common in the control group. 

Comparing all four groups allowed the identification of taxa specific for a particular clinical picture. The highest abundance of phylum Proteobacteria was found in skin samples of AD patients; lower levels were observed in ADFA and the lowest in the FA and control groups (*p* = 0.02). Sequences affiliated with Streptococcaceae were most frequent in libraries derived from skin samples of healthy children, but were absent from patients with skin symptoms (AD and ADFA; *p* = 0.04), the same applied to genus *Streptococcus* (*p* = 0.039). At the OTU level, only low abundance sequences (less than 10 reads across all groups) were found to be characteristic of a particular clinical picture. 

In the feces samples, phylum Bacteroidetes was more abundant in AD children, less frequent in ADFA and absent from healthy controls (*p* = 0.043); the same was observed to orders Bacteroidales and Xanthomonadales (*p* = 0.038 and *p* = 0.03, respectively), families Xanthomonadaceae and Bacteroidaceae (*p* = 0.03 and *p* = 0.043, respectively) and genera *Stenotrophomonas* and *Bacteroides* (*p* = 0.03 and *p* = 0.041, respectively). In contrast, the abundance of genus *Enterobacter* was higher in children with no intestinal symptoms (AD and control) but lower in those with a food allergy (FA and ADFA; *p* = 0.009). Differences in OTU abundance (Table 3) were also observed. 

To identify the taxa potentially involved in AD and FA pathogenesis, the results for the control group were compared with those from all patients with symptoms either on the skin (AD + ADFA) or in the gut (FA + ADFA) (Table 4). Libraries derived from skin of AD + ADFA patients displayed higher levels of the members of the order Xanthomonadales (*p* = 0.029) and the families Xanthomonadaceae and Corynebacteriaceae (*p* = 0.029 and *p* = 0.036, respectively). In addition, in the feces-derived libraries, class Bacteroidia (*p* = 0.007), order Bacteroidales (*p* = 0.004) and family Bacteroidaceae were found to be more common in AD + ADFA than in healthy children. 

Libraries from the skin samples of children with FA (FA + ADFA) were more abundant in bacteria from the order Xanthomonadales (*p* = 0.016) and family Xanthomonadaceae (*p* = 0.016) than those from the feces samples. Differences at the OTU level were also observed: in the skin samples, OTU2 and OTU13 were more abundant among control patients than the AD and FA groups, respectively. In the feces samples, OTUs 11, 35 and 41 were more abundant in the AD group than controls, while only OTU21 was more abundant in FA patients than the controls (Table 4).

### 3.4. There Are Taxa Whose Abundance was Positively Correlated in Feces and on Skin

Positive correlations were observed between the skin and gut libraries for the genus *Enterobacter*, regardless of patient clinical status (ρ = 0.26, *p* = 0.04). Similarly, positive correlations were observed for the genus *Gemella* for all allergic children (AD + ADFA + FA) (ρ = 0.45, *p* = 0.02), and for the genera *Lactobacillus* and *Bacteroides* in the control group (ρ = 0.48, *p* = 0.03 and ρ = 0.49, *p* = 0.02, respectively). No negative correlations were observed between the skin and feces libraries.

The abundance of 13 OTUs belonging to 11 genera was positively correlated between respective skin and feces samples (Table 5). Among them, OTU2 (*Streptococcus*) and OTU31 (*Lactobacillus*, nearest species *L. gasseri*) were correlated in both the allergic and control groups. Interestingly, no negative correlations were observed. In allergic subjects, the correlated OTUs were affiliated with Gram-positive phyla (Firmicutes and Actinobacteria), while those correlated in healthy subjects included the Gram-negative taxa *Haemophilus* (Gammaproteobacteria) and *Bacteroides* (Bacteroidetes). 

## 4. Discussion

We examined the diversity and community structure of both skin and gut (feces) microbiota in young infants with allergies symptoms, using 16S rRNA gene amplicon sequencing. In addition to being the first of its type to be performed on Polish patients, the study has four key strengths. Firstly, the gut and skin microbiota were examined simultaneously and compared. Secondly, a novel analysis was performed on the skin microbiota in infants with FA. Thirdly, the study group was characterized by a range of clinical pictures, which allowed the differences in the microbiotas to be highlighted. Finally, unlike most previous studies, the participants were under six months of age; this allows the relationship between the microbiota and the development of allergy to be assessed in an early stage of life.

The bacterial communities found in human gut and skin vary considerably between individuals and with regard to developmental stages, as do views on their relationship with allergy development [13,15,16,22,26]. These differences might stem from the profile of the studied group, e.g., the number of patients, clinical picture, demographics and environmental conditions, as well as on the methodology used, such as the primer system, PCR conditions, sequencing technology and the database used for classification [51]. This variability might explain why our results disagree with many published studies.

However, similarly to other studies [3,23,26,32,52,53,54], no significant differences in bacterial alpha diversity were found between allergic and non-allergic infants, regardless of the type of allergy or compartment, i.e., skin or feces. These results suggest that, contrary to results obtained for older patients [29,55], decreased diversity does not appear to be related to allergy symptoms, even in the case of AD. This might be due to the fact that the microbial community is still developing in young infants; nevertheless, this observation indicates that neither species richness nor diversity can be used as predictors of allergy in infants under one year of age. However, in contrast to our present findings, a number of previous studies have reported significant variation in gut microbiota α-diversity between infants with FA and healthy children [25,56]. It is possible that this variation could be attributed to the differences between the groups of participants: age (10 months [25] vs four months in our study), feeding method (currently breastfeeding 39% [25] vs 59% in our study), type of allergy (IgE-mediated [25] vs IgE + nonIgE-mediated (56 and our study)), type of allergenic food (egg [25] vs milk in our study) or, finally, genetic and environmental difference (China [56] vs Europe in our study).

We observed a significant correlation between fecal bacterial community structure and allergy type, even though the communities varied greatly within one group. This result is similar to those obtained previously [3,12]. Interestingly, both skin and fecal microbial communities differed significantly between the AD and ADFA groups, suggesting that these two clinical conditions differ in their immunological background; this difference is known to influence the microbiome, and vice versa [13,15]. Skin communities also differed, suggesting that assessing skin swabs could be a diagnostic tool for particular phenotypes of allergy. It could be possible to devise a classifier (e.g. using RandomForest framework) [57] to predict the type of allergy based on community structure. Such a tool could be useful in clinical practice, aiding in diagnosis. However, this will require further studies based on larger numbers of patients, i.e., providing greater statistical power.

Some of the taxa whose abundance was found to differ between allergic and healthy infants have previously been found to be related to allergies, although the direction of these relations varied. We found that the abundance of Bacteroidales and their member taxa, down to the level of genus (*Bacteroides* and *Parabacteroides*) in the libraries derived from feces, was related to certain types of allergy. This relationship might be driven by the production of the epithelial barrier-breaching agent phenol/*p*-cresol, which is known to be produced by the members of the Bacteroidaceae [58]. Previous studies have noted an increase in Bacteroidales members correlated with various types of allergy [53,59,60]. On the other hand, contrary to our present findings, a number of studies have reported a higher abundance of Bacteroidetes in healthy individuals [11,20,56]; however, these studies employed groups comprised of participants from different countries (Canada, Taiwan, China) and at different ages (thirteen months [20] vs three months, twelve months [11], three months [56], four months in our study).

Bacteria of the order Xanthomodales, particularly those of the genus *Xanthomonas*, are frequently found on healthy skin, albeit at a low abundance [61]. In the present study, they were associated with skin communities from allergic patients: the first such report. The abundance of *Xanthomonas* was found to increase after successful treatment of AD with emolients [62]. Although the precise role of these bacteria in skin dysbiosis remains unclear, it is possible that their keratinolytic properties may be involved. Alternatively, they might participate in shaping the microbiome structure via microbe–microbe interactions. 

Many authors have indicated that Staphylococcus plays an important role in the relationship between AD and skin colonization [29,32,63]. Kong et al. reported an increase in the number of Staphylococcus, including both *S. aureus* and *S. epidermidis*, in patients aged 2–15 years with AD exacerbation, and that topical skin treatments increased the proportion of Streptococcus, Propionibacterium and Corynebacterium [29]. Similarly, our findings indicated that the children with AD were characterized by a smaller proportion of Streptococcus than the controls. 

Assuming the existence of the gut–skin axis in humans, i.e., changes in the skin microbiome that have been found to modulate the gut microbiome [64], and changes in skin physiology that appear to induce changes in the gut microbiota [65], we hypothesize that microbiota of the skin would influence that of the gut and vice-versa. This relationship was examined in the present study.

It seems likely that the gut microbiome influences the health status of skin [66,67,68,69], particularly in the pathogenesis of AD [17]. Certain organisms, such as *Escherichia*/*Shigella* or *Veillonella*, were found to be more abundant in the feces of AD patients, while others (*Streptococcus*, *Haemophilus*) were more prominent in those of healthy subjects [69]. 

Our observations suggest that gut dysbiosis might be involved in the development of atopic skin inflammation, even though different organisms were differentially abundant in feces and skin libraries. Similar observations have been reported previously [23,33,35,54,66,69]. 

In spite of the great variability in bacterial communities observed in our samples, we found certain bacterial taxa to be characteristic of particular clinical conditions, and identified a positive correlation between the abundance of certain taxa (*Gemella*, *Lactobacillus*, *Bacteroides*) in the skin samples with those in the fecal samples. This observation, made for the first time, supports the skin–gut axis hypothesis [16,17,18,64,66].

Certain taxa found to be correlated between compartments in the present study have previously been noted as having an influence on allergy, e.g., *Bacteroidetes* [53] and lower taxa, particularly the genera *Bacteroides* and *Parabacteroides* in the intestines [60,70] or the genus *Staphylococcus*, particularly *S. aureus*, in the skin [29,32,63]. The taxa that correlated between compartments differed depending on the clinical picture, which could be explained by two factors: (i) transmission of microorganisms from the skin to the guts and (ii) selection by that immune system. Indeed, Beijerinck notes, “everything is everywhere, but the environment selects”, or rather, microbial communities are shaped by a combination of environmental filtering and stochasticity [71,72]. Therefore, our observed correlation indicates that selection operated similarly in the compartments in question. 

Hence, we hypothesize that the key factor affecting the community assembly of the skin and feces is the immunological profile of the host. If the organisms co-vary, then the immune system exerts similar pressure on them in different compartments, and their abundance depends on the initial load. As the sets of strains differ between healthy and allergic children, it is possible that the two groups demonstrate different immune responses. Such correlations may well be of practical importance and serve as indicators of the patient’s state of health. Interestingly, although the correlated organisms did not predominate, they were nevertheless found to be involved in allergy pathogenesis [73,74,75].

Our findings indicate that the abundance of the genus *Gemella* was positively correlated between the feces and skin communities in all allergic children, but not in healthy ones. No similar observation was found in the literature, but some relationships between *Gemella* and allergic inflammation seems to be possible. Taylor et al. [73] report a positive correlation between *Gemella* and sputum eosinophil percentage in patients with asthma. 

One of the OTUs whose abundance was correlated between the gut and the skin was *Bifidobacterium scardovii*; however, this was only observed in the allergic group. This could be explained by the fact that although most of the bacteria belonging to the genus *Bifidobacterium* have been recognized as beneficial to human health, *Bifidibacterium scardovii* is the one of five strains of the *Bifidobacterium* which are not—its presence has been associated with clinical conditions such as urinary infections [76]. 

Among the other OTUs with a positive correlation between fecal and skin microbiota in the allergic patients was *Corynebacterium nuruki*. Generally, *Corynebacteria* are believed to have a rather positive impact on human health [77,78,79]; however, *Corynebacterium kroppenstedtiian* has been associated with the microbiome of lesioned skin in AD patients [75] and the abundance of the genus *Corynebacterium* has been associated with chronic rhinosinusitis [74]. A novel finding in the present study was that OTU20, identified as *Rothia mucillaginosa*, also demonstrated a positive correlation between the skin and feces of allergic patients. Surprisingly, a positive correlation was also demonstrated for OTU 38 and 92, belonging to the genus *Lactobacillus*, which is known to bestow a protective effect against allergy [80]. 

Despite its strengths, our study has also some limitations. Most importantly, the group was quite small. This is particularly the case for the AD group, which can be attributed to the selection bias associated with recruiting the patients in an academic center specialized in FA management. In addition, despite the wealth of experience of the participating center, FA remains difficult to diagnose in such young infants; therefore, continuation of the study is planned to confirm our findings by diagnosing the children when they are older. The study group was also heterogeneous with respect to certain important characteristics, such as breastfeeding, introduction of solid food or probiotic intake; however, no statistically significant differences were found between the study and control groups with regard to these variables. It was also heterogeneous with respect to the severity of symptoms at the time of sample collection. Due to the non-specificity and diversity of allergy symptoms in such young infants, it would be very difficult to collect a group with identical clinical parameters. 

Another weakness of the study lies in the selection of primers; they could be not well-suited for both compartments, and some organisms, such as *Staphylococcus*, might not be well classified. Skin microbiota is usually assessed based on the V1-3 region and it is not encouraged to sequence V4 only [81]. On the other hand, our system generated a long fragment (450 bp), allowing for classification down to the species level for most organisms. It was also found that the V3-V4 region yielded better results in terms of diversity recovered than the V1-V2 region [82]. Finally, we collected swabs from healthy skin because not all children were in the acute phase of AD at the time of enrollment; however, we think that this approach was justified as Leung et al. report significant differences between the microbiota of non-lesioned, apparently healthy skin of children with AD and ADFA, but no such difference was observed for tissue displaying a skin rash [63]. 

Understanding the interrelationship between the skin and the gut may allow the design of new treatment models for skin diseases based on the manipulation of the gut microbiota, and conversely, for gastrointestinal diseases through the manipulation of skin microbiota. Moreover, further research on the microbiome may allow the determination of specific patterns of bacteria in multiple body compartments, which are prognostic factors for the development of allergies, and various clinical symptoms (AD, FA). They can also lead to the selection of specific probiotics for treating allergic conditions, either applied to the skin or applied enterally; our analysis suggests that *Acinetobacter* spp., *Haemophilus haemolyticus*, *Bacteroides ovatus*, *Schaalia odontolytica* and *Actinomyces graevenitzi* may be potential candidate bacteria for such treatment, their numbers being correlated with the feces and skin of healthy children. 

## 5. Conclusions

Although the gut and skin microbiota of infants with early symptoms of allergy demonstrate similar α-diversity to those of healthy infants, some differences in β-diversity and the abundance of some bacteria can be seen, which may depend on the phenotype of the allergy. Moreover, the gut and the skin microbiota appear to be related to one another; however, this relationship may differ between patients with allergies and those without. These correlations might serve as indicators of the allergic state of the host. 

## Figures and Tables

**Table 1:** Inclusion and exclusion criteria.

Group	Inclusion Criteria	Exclusion Criteria
Study group	age: 0–6 months and strong suspicion of FA and/or AD	immune deficiencies; autoimmunological disease skin infections other than atopic dermatitis (e.g, b scabies) lactose intolerance, non-specific intestinal inflammation, celiac disease cancer chronic respiratory tract diseases genetically determined diseases (e.g., cystic fibrosis, metabolic diseases) birth defects known bacterial, viral or fungal infection antibiotic treatment, either at the present time or in the previous month (Use of systemic antibiotics four weeks before study).
Control group	age: 0–6 months and healthy children, without symptoms indicating allergy, who were recruited among patients with suspected infection (UTI-urinary tract infection) or urinary tract disorders (CAKUT-congenital anomalies of the kidney and urinary tract), but which have not been confirmed, or children with FGIDs (functional gastrointestinal disorders), that resolved following standard treatment of symptoms and / or with a negative result for elimination tests or oral challenge.	

**Table 2:** Characteristics of the study and control groups.

Parameter	Study Group *n* = 59 (100%)	FA *n* = 16 (100%)	ADFA *n* = 38 (100%)	AD *n* = 5 (100%)	Control Group *n* = 28 (100%)	*p*
Sex, *n* (%)						
female	24 (40.7)	6 (37.5)	16 (42.1)	2 (40)	12 (42.9)	>0.05
male	35 (59.3)	10 (62.5)	22 (57.9)	3 (60)	16 (57.1)	
Age at specimen collection (weeks) mean ± SD	15.56 ± 7.00	16.63 ± 6.72	15.92 ± 7.43	16.6 ± 8.39	14.29 ± 6.52	>0.05
Weight at birth (g), mean ± SD	3495 ± 548	3518 ± 493	3571 ± 480	3588 ± 228	3360 ± 686	>0.05
Mode of delivery, *n* (%)						
Vaginal	43 (72.9)	12 (75)	27 (71.1)	4 (80)	16 (57.1)	>0.05
C-section	16 (27.1)	4 (25)	11 (28.9)	1 (20)	12 (42.9)	
Time of birth, Hbd, mean ± SD	39.3 ± 1.9	39.6 ± 1.7	39.5 ± 1.2	40 ± 0.7	38.7 ± 2.6	>0.05
Apg scale, points, mean ± SD	9.6 ± 1.2	9.6 ± 0.8	9.7 ± 0.6	10 ± 0	9.2 ± 1.9	>0.05
Mode of feeding at entry, *n* (%)						
Exclusively breastfeeding	28 (47.5)	9 (56.3)	18 (47.4)	1 (20)	13 (46.4)	>0.05
Mixed	7 (11.0)	2 (12.5)	4 (10.5)	1 (20)	3 (10.7)	
Milk formula	24 (40.7)	5 (31.3)	16 (42.1)	3 (60)	12 (42.9)	
Breast-feeding, *n* (%)						
No, never	8 (13.6)	1 (6.3)	1 (2.6)	1 (20)	5 (17.9)	>0.05
Yes, currently	35 (59.3)	11 (68.8)	12 (57.9)	2 (40)	16 (57.1)	
Solid food intake, Yes, *n* (%)	11 (18.6)	2 (12.5)	8 (21.1)	1 (20)	8 (28.6)	>0.05
Place of living *n* (%)						
non-rural	56 (94.9)	15 (93.8)	36 (94.7)	5 (100)	28 (100)	>0.05
rural	3 (5.1)	1 (6.3)	2 (5.3)	0	0	
Average living space per person, (m^2^) mean ± SD	24.6 ± 14.1	27.4 ± 17.9	24.4 ± 13.1	29.2 ± 19.3	22.4 ± 12.3	>0.05
Comiss score, points mean ± SD	12.71 ± 3.2	12.73 ± 1.54	13.62 ± 1.96	6 ± 0.65	5.63 ± 2.74	<0.00001
Parent’s age, years						
Mother	28.9 ± 4.6	29 ± 4.7	30.1 ± 3.9	29 ± 5.1	27.29 ± 4.9	>0.05
Father	31.7 ± 5.2	32 ± 5.4	32.37 ± 4.7	31.2 ± 4.1	30.6 ± 5.9	
Parent’s education, *n* (%)						
Mother Basic	6 (10.2)	4 (25)	1 (2.6)	1 (20)	6 (21.4)	0.03
Secondary	19 (32.2)	4 (25)	12 (31.6)	3 (60)	12 (42.9)	
Higher	34 (57.6)	8 (50)	25 (65.8)	1 (20)	10 (35.7)	
Father Basic	10 (16.9)	5 (31.3)	5 (13.2)	0	7 (25)	>0.05
Secondary	23 (39)	5 (31.3)	15 (39.5)	3 (60)	14 (50)	
Higher	26 (44.1)	6 (37.5)	18 (47.4)	2 (40)	7 (25)	
Atopy in family, *n* (%)						
Yes	43 (72.9)	13 (81.3)	28 (73.7)	2 (40)	15 (53.6)	>0.05
Mother	29 (49.2)	10 (62.5)	18 (47.4)	1 (20)	6 (21.4)	0.025
Father	19 (32.2)	4 (25)	14 (36.8)	1 (20)	6 (21.4)	>0.05
Siblings	14 (56.0)	4 (57.1)	10 (62.5)	0	4 (40)	>0.05
Siblings, *n* (%)	25 (42.4)	7 (43.8)	16 (42.1)	2 (40)	10 (35.7)	>0.05
Pets at home, *n* (%)						
Yes	25 (42.4)	8 (50)	15 (39.5)	2 (40)	13 (46.4)	>0.05
Dog	20 (33.9)	6 (37.5)	12 (31.6)	2 (40)	9 (32.1)	>0.05
Cat	5 (8.5)	4 (25)	1 (2.6)	0	3 (10.7)	>0.05
Tobaco smoke exposure, *n* (%)						
Mother active during pregnancy	2 (3.4)	1 (6.3)	1 (2.6)	0	3 (10.7)	>0.05
Mother during pregnancy passive	12 (20.3)	5 (31.3)	7 (18.4)	0	8 (28.6)	>0.05
Mother during lactation	1 (1.7)	0	1 (2.6)	0	3 (10.7)	>0.05
Child passive	14 (23.7)	5 (31.3)	7 (18.4)	2 (40)	10 (35.7)	>0.05
Antibiotics during pregnancy (3rd tr), *n* (%)	4 (6.8)	0	4 (10.5)	0	4 (14.3)	>0.05

*p*-value of Fisher’s exact test is given.

**Table 3:** Differentially abundant OTUs.

OTU		Abundance in Different Groups	*p*
11	Stenotrophomonas maltophilia	AD > ADFA > FA, C	0.030
21	Bacteroides	FA, ADFA > AD > C	0.030
35	Parabacteroides	AD, ADFA > FA, C	0.010
62	Rhizobium (Agrobacterium fabrum)	C > AD > FA, ADFA	0.023
85	Veilonella dispar	FA > ADFA > AD, C	0.015

*p*-value of Kruskal–Wallis test is given.

**Table 4:** Differentially represented OTUs—characteristic either for AD or FA.

Group	Skin				Feces			
	OTU		*p*	More Abundant in	OTU		*p*	More Abundant in
AD + ADFA vs C	87	Acinetobacter variabilis	0.012	AD + ADFA	11	Stenotrophomonas maltophilia	0.012	AD + ADFA
					35	Parabacteroides	0.006	
	2	Streptococcus sp.	0.044	C	41	Bacteroides dorei	0.043	
FA + ADFA vs C	13	Bradyrhizobium sp	0.027	C	21	Bacteroides	0.008	FA + ADFA
					30	Serratia marcescens	0.042	C
					68	Lactococcus lactis	0.042	

*p*-value of Kruskal–Wallis test is given.

**Table 5:** OTUs whose abundance on skin and in feces is correlated.

Infants with Allergy Symptoms				Healthy Infants			
OTU		rho	*p*	OTU		rho	*p*
2	Streptococcus sp.	0.34	0.030	2	Streptococcus sp.	0.17	0.030
14	Gemella sp.	0.45	0.002	25	Acinetobacter sp	0.72	0.0002
16	Bifidobacterium scardovii	0.35	0.020	31	Lactobacillus gasseri	0.64	0.0002
17	Corynebacterium nuruki	0.72	0	32	Haemophilus haemolyticus	0.55	0.010
20	Rothia mucillaginosa	0.45	0.002	40	Bacteroides ovatus	0.61	0.004
31	Lactobacillus gasseri	0.35	0.020	53	Schaalia odontolytica	0.46	0.030
38	Lactobacillus sp.	0.68	0	74	Actinomyces graevenitzi	0.72	0.0002
92	Lactobacillus salivarius	1	0				

Spearman’s rho and *p*-value are given.

## Data Availability

Sequence data generated during this project are available in the SRA database under BioProject no. PRJNA657878.

## Appendix A

**Table A1:** Supl. Clinical characteristic of the study group.

Parameter	Study Group *n* = 59 (100%)	FA *n* = 16 (100%)	ADFA *n* = 38 (100%)	AD *n* = 5 (100%)
Clinical symptoms, *n* (%)				
skin				
rush, erythema, xerosis	46 (77.96)	3 (18.75)	38 (100)	5 (100)
itching	0	0	17 (44.73)	2 (40)
urticarial/angiooedema	5 (8.47)	1 (6.25)	4 (10.25)	0
gastrointestinal				
colic/abdominal pain	43 (72.88)	12 (75)	29 (76.31)	2 (40)
vomiting/massive regurgitation	26 (44.06)	6 (37.5)	19 (50)	1 (20)
weight loss/poor weight gain	9 (15.25)	0	9 (23.68)	0
loose of apetite /flatulence/reflection	6 (10.16)	2 (12.5)	4 (10.52)	0
diarrhea	24 (40.60)	8 (50)	16 (42.10)	0
blood/mucus in stool	23 (38.98)	9 (56.25)	13 (34.21)	1 (20)
constipation	2 (3.38)	1 (6.25)	0	1 (20)
others				
runny nose/snuffles	9 (15.25)	5 (31.25)	4 (10.52)	0
wheezing	4 (6.77)	1 (6.25)	3 (7.89)	0
recurrent cough	2 (3.38)	1 (6.25)	1 (2.63)	0
pallor/cyanosis after ingestion	6 (10.16)	3 (18.75)	3 (7.89)	0
sweating, weakness after eating food	3 (5.08)	2 (12.5)	1 (2.63)	0
sIgE, *n* (%)				
at least >1	10 (16.94)	2 (12.5)	8 (21.05)	0
cow’s milk	0	2 (12.5)	6 (15.78)	0
egg	0	0	1 (2.63)	0
wheat	0	0	1 (2.63)	0
peanut	0	0	1 (2.63)	0
SCORAD index, points				
mean ± SD	23.77 ± 19	-	23.03 ± 17.4	29.4 ± 20.8
Atopic dermatitis severity, *n* (%)				
Mild <20 pkt	22 (37.28)	0	20 (52.63)	2 (40)
Moderate 20–40 pkt	14 (23.72)	0	13 (34.21)	1 (20)
Severe >40 pkt	7 (11.86)	0	5 (13.15)	2 (40)
AD onset (<12 weeks), *n* (%)	35 (59.32)	-	31 (81.57)	4 (80)
mean ± SD	6.4 ± 5.4	-	6.3 ± 5.3	6.6 ± 5.5
Types of CMA from gastrointestinal tract				
FPIP	19 (32.20)	8 (50)	11 (28.94)	0
FPIES	6 (10.16)	2 (12.5)	4 (10.52)	0
FGIDs	21 (35.59)	5 (31.25)	16 (42.10)	0
IgE-mediated FA	8 (13.56)	2 (12.5)	6 (15.78)	0
